# The Role of Glucose Transporters in Brain Disease: Diabetes and Alzheimer’s Disease

**DOI:** 10.3390/ijms131012629

**Published:** 2012-10-03

**Authors:** Kaushik Shah, Shanal DeSilva, Thomas Abbruscato

**Affiliations:** Department of Pharmaceutical Sciences, School of Pharmacy, Texas Tech University Health Sciences Center, 1300 S. Coulter Street, Amarillo, TX 79106, USA; E-Mails: Kaushik.Shah@ttuhsc.edu (K.S.); Shanal.Desilva@ttuhsc.edu (S.D.)

**Keywords:** glucose transporter, GLUT, SGLT, diabetes, Alzheimer’s disease, blood brain barrier, glucose metabolism

## Abstract

The occurrence of altered brain glucose metabolism has long been suggested in both diabetes and Alzheimer’s diseases. However, the preceding mechanism to altered glucose metabolism has not been well understood. Glucose enters the brain via glucose transporters primarily present at the blood-brain barrier. Any changes in glucose transporter function and expression dramatically affects brain glucose homeostasis and function. In the brains of both diabetic and Alzheimer’s disease patients, changes in glucose transporter function and expression have been observed, but a possible link between the altered glucose transporter function and disease progress is missing. Future recognition of the role of new glucose transporter isoforms in the brain may provide a better understanding of brain glucose metabolism in normal and disease states. Elucidation of clinical pathological mechanisms related to glucose transport and metabolism may provide common links to the etiology of these two diseases. Considering these facts, in this review we provide a current understanding of the vital roles of a variety of glucose transporters in the normal, diabetic and Alzheimer’s disease brain.

## 1. Introduction

Glucose is a vital metabolic fuel for all mammalian cells. Under normal physiological conditions cells are entirely dependent on a continuous supply of glucose and many other blood-borne nutrients. This process is mainly limited at the cellular level by the presence of impermeable cell membranes and at the tissue level by a barrier between tissues and their blood supply. In some cases this barrier prevents passive diffusion of glucose and other nutrients into and out of tissue and cells. As an alternative, glucose is transferred across the cell membranes and tissue barriers by a specific saturable transport process involving members of two different classes of glucose transporters, sodium-independent glucose transporters (facilitated transport; GLUT) and sodium-dependent glucose transporters (secondary active transport; SGLT), each with different kinetic properties. Most cells express a variety of glucose transporters and the pattern of expression in different tissues is related to specific metabolic requirements. Compared to peripheral organs availability of glucose and other nutrients to neural tissues is limited by the dynamic restrictive properties of the blood-brain barrier (BBB), which is formed by distinct characteristics (tight junctions, lack of fenestrations and pinocytic activity). Due to the restrictive permeability of the BBB and the relative lack of local brain carbohydrate stores (glycogen, mostly in astrocytes), the CNS relies heavily upon BBB expression of transporters for the delivery of key nutrients and solutes to the brain.

Also recent accumulated evidence suggests that lactate produced by astrocytes is another key energy substrate preferentially utilized by neuronal cells [1,2]. It is believed since lactate transport across BBB is minimal [3], release of glutamate during neuronal activation stimulates glucose uptake and production of lactate within brain parenchymal astrocytes and release is coupled to neuronal activity. Most astrocytic lactate is obtained from anaerobic metabolism of glucose [4] and small amounts from glycogenolysis [5]. Importantly, in order for astrocytes to produce lactate, glucose has to be transported across the BBB and the astrocytic membrane via transporters. Additionally, ketones can be considered an alternative brain fuel since they can be utilized by brain capillaries [6] and astrocytes [7]. During the first two postnatal weeks the brain uses glucose and ketones as energy substrates. In early postnatal lifespan (suckling stage) compared to an adult, BBB permeability to ketones is very high, and glucose transport capacity is low. Conversely at the end of suckling period, the brain relies heavily on glucose as energy substrate, owing to decreased permeability of ketones by 62% and an increase in the rate of glucose transport by threefold. Since in adult brains the fatty acids transport across the BBB is extremely slow [8], they do not provide carbon to the tri-carboxylic acid cycle or the precursor for lactate production [9].

Given that glucose is the major obligate energetic fuel of brain tissue, the availability of glucose and its transport into the brain across the BBB and into individual brain cells plays a key role in normal physiological function and energy metabolism. Thus, it is essential to understand the role of altered glucose transport in brain cells and at the BBB in brain diseases and the pathogenesis of complications associated with diseases such as diabetes.

With the increasing prevalence of diabetes worldwide, the World Health Organization predicts diabetes-related deaths will double between 2005 to 2030. Currently diabetes is the seventh leading cause of death in United States (US). 28.8 million people in the United States have diabetes with estimated health care cost of $174 billion. Similarly, Alzheimer’s is the sixth leading cause of death in the US. 5.4 million people in the US are living with Alzheimer’s. The recent preclinical and clinical link between reduced glucose metabolism, diabetes mellitus and Alzheimer’s disease [10–15] warrants discussion of these translationally relevant topics. The present review will focus primarily on glucose transport and glucose transporter expression in the brain, and its consequences in different pathological conditions such as diabetes mellitus and Alzheimer’s disease. The physiologic consequences of facilitative or sodium-dependent processes in disease states such as these will also be discussed.

A series of reviews on many of the glucose transporters, including GLUT and SGLT, have been published [16,17]. The GLUT isoforms, of which 12 have been identified, belong to *SLC2A* hexose transport family. The six identified SGLT isoforms belong to the *SLC5A* sodium-dependent co-transporter family. The GLUT family of transporters contains membrane spanning glycoproteins with 12 transmembrane domains, a single *N*-Glycosylation site. GLUT family proteins transport glucose bidirectionally based upon a concentration gradient. Different isoforms show relative affinity for glucose suggesting adaptation to the different metabolic requirements of each cell [18]. Compared to GLUT, the SGLT family of co-transporters contains membrane spanning monomer proteins with 14 transmembrane domains and a single *N*-Glycosylation site. SGLTs transport glucose and galactose against a concentration gradient with simultaneous transport of Na^+^ ions [19]. Asymmetric luminal expression of SGLT1-2 has mainly been shown to be involved in glucose (re)absorption in intestine and kidney.

## 2. Neurovascular Unit: Glucose Transporters and Transport

**Figure 1:**
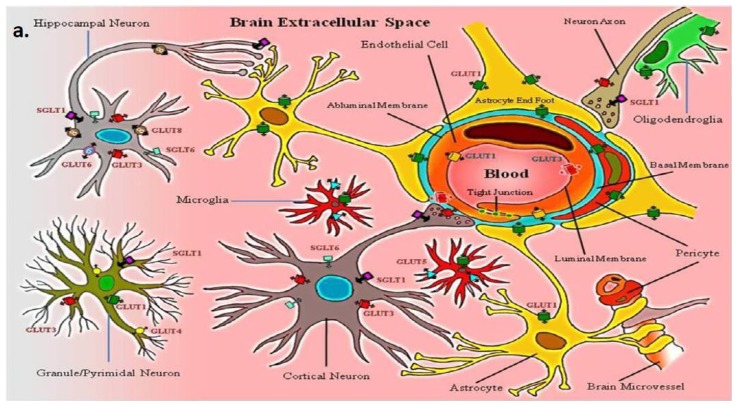

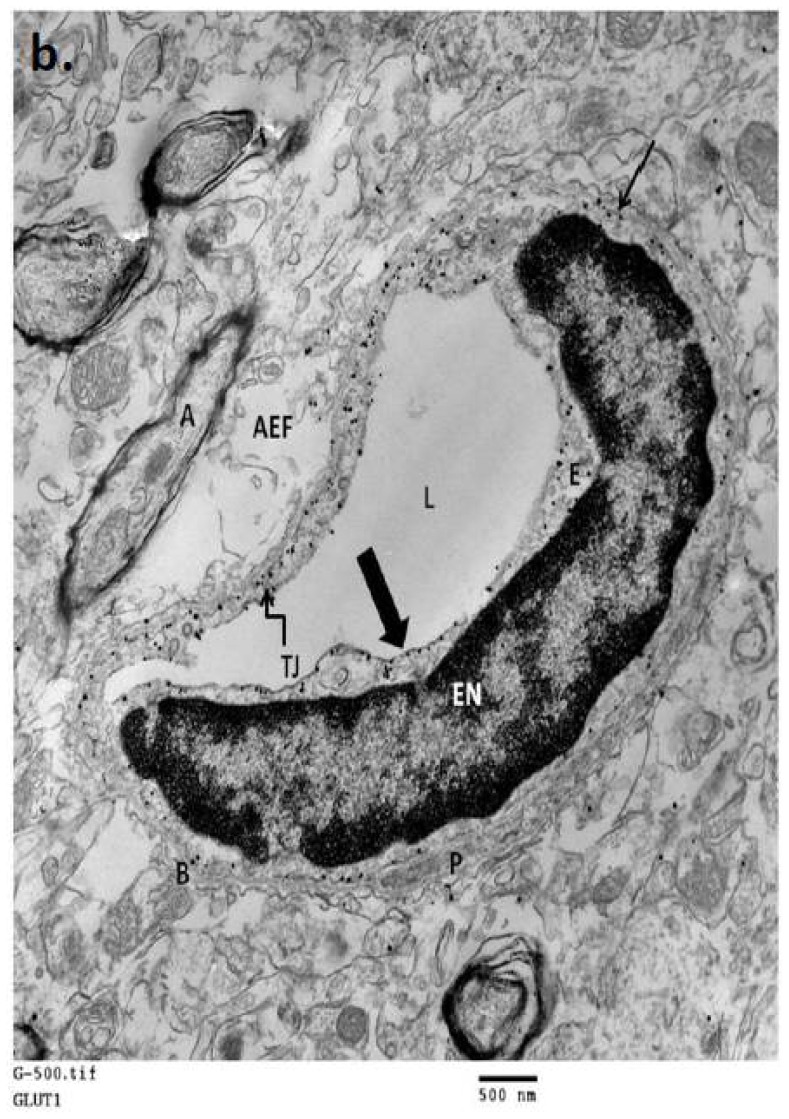
(**a**) A schematic representation of the cellular localization of glucose transporters (GLUTs and SGLTs) in mammalian neurovascular unit; (**b**) An electron micrograph of normal mouse brain neurovascular unit showing polarized expression of GLUT1 transporter in endothelial cells. L-lumen (blood side); E-endothelial cell; EN-endothelial nucleus; P–pericyte; B-basement membrane; TJ-tight junction; AEF-astrocyte end foot; A-myelinated axon; Thick arrow, shows luminal membrane with 6 nm silver enhanced immunogold labeled GLUT1 protein; Thin arrow, shows abluminal membrane with immunogold labeled GLUT1 protein. (Immunogold labeling and micrograph generated in the Abbruscato Lab.)

Several glucose transporters have been identified in the brain [20–23]. Expression of different isoforms of both the GLUT and SGLT families has been shown to be cell type specific [22,24,25] (Table 1 and Figure 1a). Among the membrane transport proteins shown to be expressed in various tissues, GLUT1 is the most well characterized and studied. The GLUT1 gene is located on human chromosome 1 (1p35-31.3) [26], encoding a transport protein that is mainly expressed at the BBB. It is known to be a key regulator of glucose transport into and out of the brain across the BBB acting to maintain central nervous system homeostasis. It is also highly expressed in proliferating cells of the early developing embryo, cardiac muscle, human erythrocytes and astrocytes [27]. GLUT1 is found in the cells forming blood-tissue barriers [28], such as the choroid plexus and blood-spinal cord barrier, and the ependymal lining of the cerebral ventricles [29,30]. In addition, to glucose GLUT1 also transports galactose and ascorbic acid [31,32].

Glucose enters the brain extracellular space from blood via the GLUT1 transporter present on both the luminal and abluminal membranes of the BBB endothelial cells (Figure 1a,b). Many studies have confirmed the presence of GLUT1 protein in brain microvascular endothelial cells as well as in astrocytes [33–35], which are in close proximity to brain endothelial cells. GLUT1 is localized to both luminal and abluminal membranes of the BBB endothelial cells with the ratio of 1:4 respectively [36–39], and approximately 40% of the total cellular GLUT1 resides in intracellular membrane [40]. GLUT1 can function at less than its maximum functional capacity under normal conditions, making it viewed as less of a rate limiting factor for brain function [41]. In contrast to glucose transport, some believe that glucose phosphorylation by hexokinase is the rate-limiting step in the process of brain energy metabolism [9].

GLUT1 proteins present at BBB endothelial cells are heavily glycosylated, high molecular weight (55 kDa) isoforms compared to GLUT1 proteins (45 kDa) present in astrocytes [42]. However, the effect of differential glycosylation on protein transport kinetics has not been well established. Circulating glucose concentrations regulate the endothelial GLUT1 protein concentration [43] as well as expression, which appear to be under both transcriptional and post-transcriptional control [44,45]. Recently, GLUT1 has been shown to be differentially phosphorylated at the luminal and abluminal membranes of BBB endothelial cells suggesting possible conformational changes leading to transport activity modulation [46]. Additional glucose transporters have also been identified at BBB endothelial cells with lower levels of GLUT3 [47–51], GLUT4 [52] SGLT1 [53] and SGLT2 [50] compared to GLUT1 protein levels in human as well as other species. Insulin sensitive GLUT4 expression has also been shown to be at the BBB of the ventromedial hypothalamus suggesting its role in brain sensing of blood glucose concentration [52]. However, complete functional characterization of these transporters at the BBB needs to be addressed. Embryonic lethality of the homozygous mutant of GLUT1 as well as GLUT3 suggests its important role in overall developmental processes [54,55]. Importantly, heterozygous GLUT1 mouse shows brain abnormalities with reduced brain size [54], whereas heterozygous GLUT3 mouse shows normal brain size with cognitive abnormalities [56], further suggesting dominant role of GLUT1 at the BBB in glucose transport.

**Figure 2:**
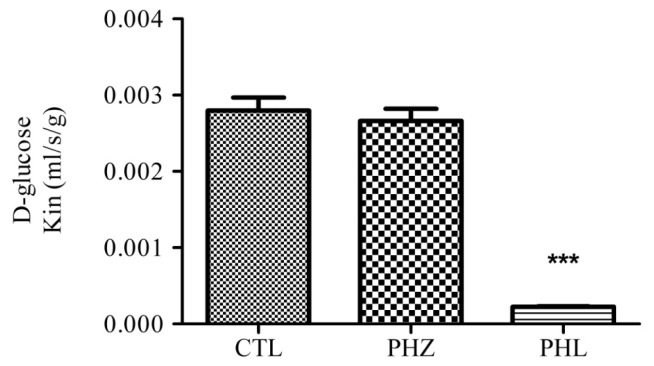
[^3^H] D-glucose transport (*K*_in_; mL/sec/g) in the control brains of C57BL/6J mice was measured by an *in situ* carotid perfusion for 20 s at 2.5 mL/min. The bicarbonate buffer solution containing [^3^H] D-glucose (25 nmol/L) and cold D-glucose (0.5 mmol/L) was perfused with (or without; controls, CTL) inhibitors Phlorizin (PHZ; SGLT inhibitor, 50 μM) and Phloretin (PHL; GLUT inhibitor, 50 μM). Data are means ± SEM of 6 to 9 animals, analyzed using one way ANOVA test Post hoc Bonferroni’s Multiple comparison ** *p* < 0.01, *** *p* < 0.001.

Although, a few studies have suggested luminal expression of SGLT1 at BBB endothelial cells [53,57], recently Yu *et al*. [58] demonstrated no functional SGLT protein at the BBB endothelial cells when SGLT specific substrate uptake was measured in rat. Additionally, in our lab we have observed no functional SGLT role in glucose uptake at the BBB in mice using the *in situ* brain perfusion technique to quantitate the blood to brain transport of D-glucose with or without inhibitors for GLUT (Phloretin) and SGLT (Phlorizin) (Figure 2). However with reference to stroke, recent work in our laboratory has determined that SGLT does play a role in the blood-to-brain movement of glucose during *in vivo* conditions of ischemia-reperfusion and *in vitro* conditions of hypoxia-aglycemia, suggesting a pathophysiologic significance [59]. Altered functional expression of glucose transporters at the BBB in various pathological conditions are discussed later in this review.

**Figure 3:**
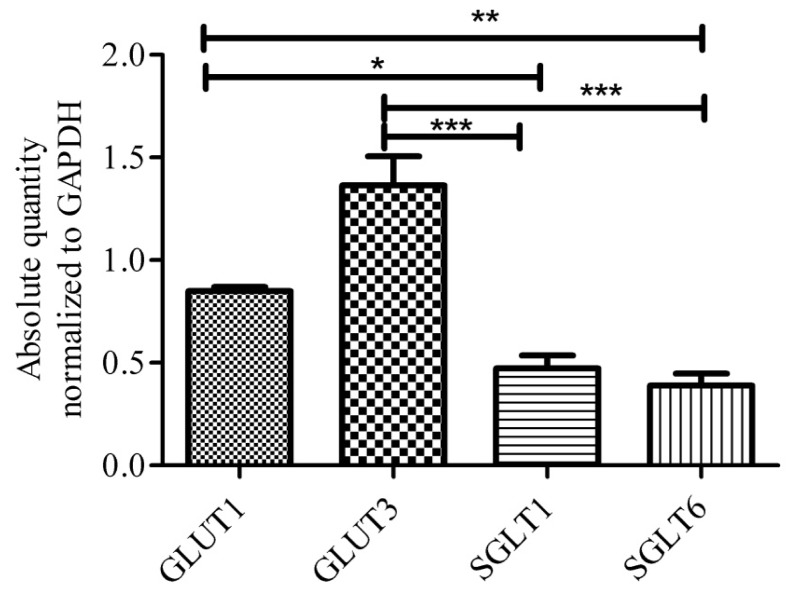
Expression of *GLUT1*, *GLUT3*, *SGLT1* and *SGLT6* mRNA of whole mouse brain tissues. Transcripts of glucose transporters per 0.75 μg of total RNA. Data shows normalized absolute quantity of transporter using qPCR SYBR technique. Data respresents mean ± SEM of 7–8 animals, analyzed using one way ANOVA test Post hoc Bonferroni’s Multiple comparison * *p* < 0.05, ** *p* < 0.01, *** *p* < 0.001.

Once glucose enters the brain extracellular space, it is rapidly taken up by the different brain cells. Specifically, neurons express GLUT3, a high “affinity” and high capacity glucose transporter compared to GLUT1 [60–62]. Under normal physiological conditions, neurons have a high energy demand. GLUT3 localization, therefore appropriately correlates with neuron function in the neuropil, along both dendrites and axons [63]. In addition to GLUT3, functional SGLT1 is shown to be constitutively expressed in different brain regions including hippocampus and cerebral cortices of rat [58]. Conversely, recent studies showed no mRNA transcript for *SGLT1* in different human brain regions, in fact, this study observed high levels of *SGLT6* and sodium-myoinositol cotransporter *(SMIT)* transcript in human brain [64]. Both SGLT6 and SMIT are known to transport myo-inositol as well as glucose, but their functional significance in neurons is yet to be evaluated. In our lab we have also observed the expression of *SGLT1* and *SGLT6* transcripts in mouse brain, however, the levels of *SGLT1* and *SGLT6* transcripts are lower compared to *GLUT3* and *GLUT1* (Figure 3). Others have suggested the expression of insulin sensitive GLUT4 in discrete subsets of neurons [65–68]. Recent studies are also proposing a possible role for GLUT8, which is a hormonally-regulated glucose transporter in hippocampal and cerebellar neurons located in intracellular compartment, but no definitive cell surface localization and function is yet known [65,69–77]. The fructose transporter, GLUT5 has also been shown to be expressed in human and rat microglial cells [78]. GLUT5 has very low affinity for glucose; the functional significance of this fructose transport in microglia is also not yet known. Moreover, no other glucose transporters have been identified to be present in microglia [79].

The presence of insulin-insensitive glucose transporters in the brain and the regulation of glucose transport by glucose concentration itself may play an important role in the pathogenesis of diseases where blood glucose levels are perturbed chronically. With these newly identified transporters in the brain, a complete characterization is warranted in order to understand the role of each glucose transporter in brain physiological function relative to each other as well as each of their contributions to the pathogenesis of brain diseases.

## 3. Diabetes: Glucose Metabolism and Glucose Transporters in the Brain

Diabetes is characterized by high levels of blood glucose resulting from defects in insulin production, insulin action or both. Patients with type 1 (insulin-dependent) or type 2 (insulin-independent) diabetes often develop secondary complications. Mostly these secondary complications arise in the organs that exhibit insulin-independent glucose uptake. Pathophysiological complications associated with diabetes mainly attributed to increased sorbitol production, oxidative-nitrosative stress, endogenous antioxidant depletion, enhanced lipid peroxidation, metabolic changes, and altered hormonal responses [80]. However, the major mechanism that precedes all the above is increased intracellular glucose and the downstream metabolic fate. Thus, it is crucial to understand brain glucose transporter function, expression and regulation, which is often a rate-limiting step in the pathogenesis of the complications associated with a diabetic brain. Experimental diabetes is manifested by large increases in glucose levels in blood and body tissues, including brain. In experimental diabetes models, hyperglycemia increases neuronal glucose levels up to fourfold [81–94]. Recent reports using the streptozotocin (STZ) induced-diabetic rat model have shown a significant increase in brain glucose concentration as measured by NMR spectroscopy. They have also shown no change in dependence of hippocampal glucose on plasma glucose levels suggesting no alteration in BBB glucose transporters in these diabetic animals [95].

Over the decades a number of reports have been published on the effects of experimental diabetes on glucose transporter expression, specifically GLUT1, in cerebral cortex and cerebral microvessels. The results of these reports remain mixed as they fail to correlate glucose transport function with expression of the transporter. Investigators have reported a decrease [96–99] or no change [100–102] in GLUT1 function in cerebral microvessels of diabetic rats. The first report showing a decrease in blood-brain glucose transfer in rats with chronic hyperglycemia was published in 1981 using an intravenous bolus infusion method [103]. Later, findings of this study were subsequently confirmed by several investigators using different experimental approaches [97,104–106]. Differences in the techniques utilized and inherent problems in calculating glucose transfer rates using these techniques were detailed in peer review by Pelligrino *et al*. [101]. However, to avoid some of the uncertainties inherent in the BUI (brain uptake index) method, Pardridge *et al*. [97] used an *in situ* internal carotid artery infusion technique to estimate BBB glucose transport in chronic hyperglycemia. The method allowed for simultaneous measurements of glucose extraction and cerebral blood flow in the same animal. The authors concluded that there was no change in brain vascular space, a 44% decrease in cerebral blood flow and a 44% reduction in BBB transport of glucose. It should be noted however, that changes in glucose transport measurement may be due in part to differences in steady state glucose concentration at the capillary bed in normoglycemic and hyperglycemic animals during infusion, which can further inhibit glucose influx. Similar to a published method by Cattelotte *et al*. [107], our lab performed mouse *in situ* brain perfusion to estimate BBB glucose transfer in normoglycemic and 2-week-old STZ induced hyperglycemic mice. In these animals we pre-equilibrated the endothelial cells with a known subsaturating concentration of cold glucose (0.5 mM) for 30 s and then perfused with radiolabelled ^3^H-D-glucose for 20 s. Contrary to the above mentioned studies, we did not observe changes in total glucose influx, cerebral blood flow or brain vascular volume of animals with hyperglycemia compared to normoglycemia. In addition, we did not observe changes in glucose transporter affinity (*K*_m_) or maximum rate of transfer (*V*_max_) for glucose, suggesting no change in glucose transport across the BBB in experimental diabetes [108]. Some investigators have reported increased levels of *GLUT1* mRNA in cerebral microvessels in the db/db type-II diabetes mouse model despite normal GLUT1 protein concentrations [102], suggesting a diabetes-induced defect in translation of the transporter. Furthermore, differential regulation of transporters in the microvasculature of different organ systems has been suggested from observing reduced retina GLUT1 protein expression in its microvasculature with no change in GLUT1 expression in cerebral microvessels in 2-week or 2-month old STZ-induced diabetic rats [109]. Converse to the findings by Lutz *et al*. [110], insulin normalizes *GLUT1* mRNA but not protein in 1-week-old STZ-diabetic rats. A recent study suggests an inverse correlation in protein and mRNA levels with different blood glucose concentrations maintained with insulin in these animals [111].

Duelli *et al*. [112] have shown increased local cerebral glucose utilization and decreased GLUT1 protein density in 3-week-old STZ-induced diabetic rats. They also showed a positive correlation of local densities of GLUT1 and GLUT3 with local cerebral glucose utilization during non-diabetic conditions as well as during chronic hyperglycemia. Nevertheless, these findings should be re-evaluated based on the current knowledge of additional transporters (GLUT8, GLUT4, SGLT1, SGLT6), which may contribute to glucose transport in both normal and diabetic brains, specifically neurons. Besides, GLUT4 levels have been shown to be increased in the cerebellum of db/db mice and decreased in STZ induced diabetic rats, suggesting insulin sensitive GLUT4 expression in two different models of diabetes [113]. Similar to peripheral tissues, insulin stimulated translocation of GLUT4 to the plasma membrane in rat hippocampus has been shown to be phosphatidylinositol 3-kinase dependent [114]. Additional contributions by this transporter in complications associated with two different (insulin-dependent and non-insulin dependent) chronic hyperglycemic conditions should also be investigated.

On the other hand, similar reports in humans showing changes in brain glucose concentration, utilization and metabolism are sparse. Likewise, discrepancies still exist in understanding the effects of diabetes on brain glucose concentrations, whereby some studies show diabetes causes an increase [115,116] and others a decrease [117]. Also many investigations have shown no change in brain glucose transfer and metabolism in both well controlled and poorly controlled insulin-dependent diabetes [118–121]. Criego *et al*. [116] demonstrated increases in steady state brain glucose concentration in type 1 diabetic patients with hypoglycemia unawareness which could result from adaptive changes in glucose transporter at the BBB or metabolism in the brain due to recurrent hypoglycemic episodes. A recent study by Henry *et al*. [122] demonstrated no change in the cerebral oxidative metabolic rate of glucose with ^13^C-NMR in type 1 diabetic patients with hypoglycemia unawareness compared to normal control subjects, further supporting the increased brain glucose concentration may be a result of increase in glucose transporter (s) at the BBB. Similar studies examining brain glucose concentration, metabolism and transport in both insulin and non-insulin dependent diabetic patients are needed to completely understand glucose induced perturbations in the brain. The observed variability in glucose transporter expression and function could be in part attributed to differences in duration of chronic hyperglycemia, severity, animal species and diabetic models used in the studies. Simultaneous observation of glucose regulatory and counter-regulatory mechanisms may provide better insight into these variable results.

## 4. Alzheimer’s Disease: Glucose Metabolism and Glucose Transporters in the Brain

As mentioned earlier, glucose transport across the BBB and/or the neuronal plasma membrane can become a rate-limiting step under diminished brain fuel conditions or in pathological situations such as Alzheimer’s disease (AD), epilepsy, dementia, ischemia and traumatic brain injury [123]. Histopathologically, AD is distinguished by the presence of extracellular amyloid plaques (APs) or intracellular neurofibrillary tangles (NFTs) in the brain [124]. It has been suggested that the degeneration of neurofibrils, which can be observed in studies which correlate dementia symptoms to the number of brain tangles [125–127], may be contributing to the state of AD [124]. Abnormally hyperphosphorylated tau, which gradually accumulates into coupled helical filaments and straight fragments, makes up the NFTs [124,128]. Furthermore, overproduction of amyloid beta peptide (Aβ) along with early onset familial AD is suggested to be a key player in AD [129]. AD is considered one of the most progressive destructive neurodegenerative diseases causing dementia and eventually can lead to death of patients [130].

AD can be classified into two types. Only 5%–10% of AD cases (type I AD), are reported to be early onset and a result of genetic abnormalities [9,131], while the rest of the cases (90%–95%) are sporadic (type II AD) [131]. A study in the early 1990s revealed that glucose -based ATP production (glucose metabolism) is declined by 50% in type II, sporadic AD, which also continues to decline throughout the progression of the disease [132] whereby a ~20% energy deficit was observed [131]. This occurred despite compensatory mechanisms of utilizing endogenous brain substances, such as glutamate and fatty acids [133–135], which can result in neurotoxic by-products like ammonia [136]. Hoyer [132] also reports that type I AD (early onset) does not display a significant loss in ATP production compared to type II AD. Also, since aging is considered an important risk factor, it is believed that altered glucose utilization and metabolism with aging is a contributing factor in type II AD. Many studies validate this hypothesis [137–140]; however, there is not much data to support glucose hypometabolism as a cause or effect in the pathogenesis of AD.

The healthy adult human brain cerebral metabolic rate of glucose is 6–7 mg/100g/min with a whole brain equivalent of 120–130 g glucose/day [9,141]. Although about 2% of the entire human body mass is composed by the brain, under basal conditions 50% of the utilization of glucose occurs in brain metabolism [142,143]. The majority of the brain glucose is converted to ATP energy for the maintenance of normal neuronal functions including cognition. In AD reduction of the brain glucose utilization differs regionally [124,144–146]. According to Hoyer [147] this variation is a cause for neurodegeneration rather than a mere consequence. Apart from glucose utilization for energy production, roughly 2%–5% of it is used in the hexosamine biosynthesis pathway (HBP), involved in production of UDP-*N*-acetylglucosamine (UDP-GlcNAc) [148]. It is a substrate for O-linked β-*N*-acetylglucosamine (O-GlcNAc) transferase (OGT) which is required for catalyzing O-GlcNacylation of Ser/Thr protein residues [143]. Interestingly, reduced O-GlcNAcylation has been linked with changes in glucose uptake and metabolism [124,149,150]. A proof-of-concept was reported by Liu *et al*. [143] which showed that downregulation of O-GlcNAcylation, by using a small hairpin RNA to knockdown the O-GLcNAc transferase led to increased hyperphosphorylation of tau in HEK-293 cells. They also reported that HBP inhibition led to the reduction of O-GlcNAcylation and elevated tau phosphorylation, which mirrors the fasting induced reduction in glucose metabolism in rodent brains but not in phosphatase 2A inhibited rat brains. This suggests that the downregulation of both O-GlcNAcylation and phosphatase 2A protein may lead to the progression of AD.

There is an ongoing debate if age is necessarily associated with lower glucose metabolism in the brain, as advancing age is believed to alter the systemic glucose utilization process, which may hinder brain glucose uptake [9]. Along with aging, diabetes and insulin resistance may be the two most important risk factors influencing brain fuel metabolism in AD. Though the downstream effects of reduced glucose metabolism have been shown to be major contributors to both human and rodent AD [151–154], the upstream specific molecular mechanisms have not been established. Emerging evidence from various *in vitro* and *in vivo* studies reveals that brain hypometabolism may precede the notable cognitive decline in AD and may contribute to the severity of the disease [9]. Although less than 1% of the normal brain weight is represented by brain endothelial cells, they transport roughly 10 times more glucose/min compared to their weight [155]. Therefore, it has been proposed that any dysfunction in brain endothelial mitochondria may alter glucose transport across the BBB and increase the risk of AD [9]. Thus, it is worthwhile to understand the preceding mechanisms leading to hypometabolism such as changes in glucose transporter function, impaired glycolysis and dysfunctional mitochondria in the brain.

A few reports have studied actual glucose transport in the human AD brain [156–160] using PET. These studies have shown reduced glucose transport in the most metabolic active brain regions such as cortex, hippocampus and cerebral microvessels of AD patients. Similar glucose uptake studies have also been performed in animal models of AD. For example age related reduction in *F*-fluorodeoxyglucose (FDG) uptake in various brain regions has been observed in different transgenic mice, including a triple transgenic mouse model of AD [161–164]. This observation suggests that reduced FDG uptake is an effect of transgenic proteins expressed in these mice and not vise-versa. It is believed that synaptic dysfunction, *i.e*., loss of synapse and neuronal death, is caused primarily by Aβ deposition which induces lipid peroxidation, disruption of ion homeostasis and apoptosis. The increased lipid peroxidation by Aβ deposition has been shown to reduced glucose transport and metabolism in hippocampal and cortical neurons *in vitro* [165]. In a clinical study this relationship between glucose metabolism and Aβ deposition on brain function in AD patients has been studied using the recently developed PET ligand (Pittsburgh Compound-B;PIB) which trails Aβ deposition along with glucose metabolism marker ^18^F-fluorodeoxyglucose (FDG). This study concluded that the severe Aβ deposition is not associated with a comparable decrease in brain glucose uptake and metabolism [166].

Whether deranged brain glucose uptake is due to defects in glucose transporter at the BBB or brain parenchyma has also been clinically investigated. AD patients show diminished GLUT1 and 3 expressions especially in the cerebral cortex, with significant loss of GLUT3 [167]. One such study have also shown the reduced expression of GLUT3 in the dentate gyrus [168], while another reported a significant lowering of GLUT1 expression with no difference in mRNA levels of *GLUT1* [169] in the human AD brain suggesting post-transcriptional regulation. Liu *et al*. [124] reported a positive correlation between the decreased O-GlcNAcylation and GLUT1 and 3 reduction, and a negative correlation between GLUT1 and 3 reduction and tau phosphorylation levels at multiple sites. They therefore [143] propose that defective glucose transport leads to reduced glucose metabolism, which causes abnormal tau phosphorylation and/or neurofibrillary degeneration by HBP down regulation. This ultimately results in tau O-GlcNAcylation.

One study group compared GLUT1 through 4 by using human brain tissue samples (7 AD and 7 control brains). They showed decreased protein levels of GLUT1 and GLUT3 in the AD brain which correlated to decreased O-GlcNAcylation and tau hyperphosphorylation [124]. They also witnessed a drastic increase in GLUT2 in the AD brain. Further confirming the abnormal hyperphosphorylation of tau and degeneration of neurofibrils may be directly linked to changes in glucose transporter expression, especially GLUT expression in the AD brain.

Another study by the same group using human brain samples (11 type 2 diabetes mellitus (T2DM), 10 AD, 8 T2DM and AD and 7 controls) showed that the T2DM brain had a decreased level of neuronal GLUT3 protein in comparison to the AD brain and that the decrease in O-GlcNAcylation which was observed in the AD brain was also visible in the T2DM brain [130]. They concluded that because of the observed abnormal hyperphosphorylation of tau in the T2DM brain, those patients with T2DM have an increased risk of developing AD.

Several other mechanisms have been proposed to be involved in hypometabolism associated with AD. Impaired cholinergic neurotransmission is considered to be a hallmark of AD [170,171]. It is known that acetylcholine synthesis is sensitive to the metabolism of brain glucose [172,173], and therefore reduced glucose levels may have a negative effect on cognitive function [9].

Among the different lifestyle related factors associated with AD symptoms, insulin resistance is known to be highly associated with decreasing cognition among older people [9,174,175]. Impaired insulin signal transduction is assumed [131] to cause improper brain glucose metabolism since an up-regulation of insulin receptor density was noted in AD brains similar to that seen in non-insulin dependent diabetes [176,177]. Although desensitization mechanisms of neural insulin receptors are not yet clear, it will be worthwhile to test downstream effects of this on the insulin sensitive glucose transporter (GLUT4) and its role in AD pathogenesis.

Neurons highly express insulin receptors [178–180] suggesting an important role for insulin in brain glucose and lipid metabolism including the regulation of neuronal development, memory and learning processes [180–182]. Although some studies suggest that neurons synthesize insulin [183,184], the majority of the brain insulin requirement is fulfilled by peripheral insulin which uses a saturable transport mechanism to cross the BBB [185].

Several studies done on AD patients [186–188] and rodent models of AD [189,190] support the role of dysfunctional insulin signaling in the pathogenesis of AD [180]. Liu *et al*. [180] found reduced levels and activity of many insulin-PI3K-AKT signaling pathway components which negatively correlated with the phosphorylation of tau. Also reduced insulin-PI3K-AKT levels positively correlated with reduced O-GlcNAcylation of protein. This suggests that through the downregulation of O-GlcNAcylation and/or the promotion of abnormal hyperphosphorylation of tau, neurodegeneration is a consequence of the impaired insulin-PI3K-AKT signaling pathway. The role of insulin/insulin resistance and its influence on impaired brain glucose transport and/or metabolism still remains to be established.

There is an enormous amount of clinical and experimental evidence which shows that significantly reduced brain glucose metabolism and transport, and impaired insulin signaling are present in the disease progression of AD [9]. However, with identification of more isoforms of different classes of glucose transporter families expressed in the brain, it will be interesting to see their role in glucose transport and related mechanisms in the pathogenesis of AD brain.

## 5. Future Challenges

As discussed in earlier sections, emerging evidence suggests the importance of brain glucose metabolism and transport in the complications associated with DM and neurodegenerative disorders such as AD. The identification of new isoforms of glucose transporters in the brain poses several questions that remain to be answered. In addition, there is a need to reevaluate the already established neuronal glucose transporters and their role in the physiology and pathology of DM and AD. Clinically relevant mechanistic investigations into the role and pathogenesis of diabetes mellitus, brain glucose hypometabolism and AD progression are desperately needed to improve outcomes in this highly susceptible neurodegenerative population.

## Figures and Tables

**Table 1:** The facilitative and sodium-dependent glucose transporter family.

Type	Protein (*gene*)	Sites expressed	Substrate/transports
Facilitative/Sodium-independent	**GLUT1** (*SLC2A1*)	Brain endothelial and epithelial-like brain barriers, glial cells, blood-tissue barriers, eye, pheripheral nerves, placenta, lactating mammary gland (Ubiquitous distribution in most mammalian cells)	>>Glucose, galactose, mannose, glucosamine, ascorbic acid
	**GLUT2** (*SLC2A2*)	Kidney, small intestine (epithelial cells), liver, pancreas (islets), brain (astrocytes)	Mannose, galactose, fructose, glucose, glucosamine
	**GLUT3** (*SLC2A3*)	Neurons, testis, placenta, brain endothelial cells?	Glucose, galactose, mannose, xylose, dehydroascorbic acid
	**GLUT4** (*SLC2A4*) (Insulin-sensitive)	Brown and white adipose tissue, muscle (skeletal), fat, heart (myocardium), hippocampal neurons, cerebellar neurons	Glucose, dehydroascorbic acid, glucosamine
	**GLUT5** (*SLC2A5*)	Intestine (jejunum), kidney, testis, brain microglia	Fructose
	**GLUT6** (*SLC2A6*)	Brain, peripheral and spleen (leukocytes)	Glucose
	**GLUT7** (*SLC2A7*)	Small intestine (mainly in brush border membrane-enterocytes), colon, testis, prostate, liver (associated with endoplasmic reticulum)	>Fructose, glucose
	**GLUT8** (*SLC2A8*) (Insulin-responsive?)	Blastocytes, testis, brain (neurons), muscle, adipocytes, mammary gland?	Glucose
	**GLUT9** (*SLC2A9*)	Liver, kidney (proximal tubule of epithelial cells), placenta?	Glucose, urate
	**GLUT10** (*SLC2A10*)	Liver, pancreas, heart, lung, brain, skeletal muscle, placenta	Glucose, galactose
	**GLUT11** (*SLC2A11*)	Iso-form A: Heart, skeletal muscle, kidney	Fructose, glucose
		Iso-form B: Placenta, adipose tissue, kidney	
		Iso-form C: Adipose tissue, heart, skeletal muscle, pancreas	
	**GLUT12 (*SLC2A12*) (Insulin-sensitive?)**	Heart, skeletal muscle, fat, prostrate, lactating mammary gland ?, spleen ?, breast cancer (Ductal cell carcinoma) tissue	Glucose
	**HMIT (*SLC2A13*) (co-transporter)**	Brain (neurons intracellular vesicles)	H^+^/myo-inositol

Sodium-Glucose Co-transporter/Sodium-dependent	**SGLT1** (*SLC5A1*)	Small Intestine (brush-border membrane), trachea, kidney, heart, brain (cortical, pyramidal and purkinje neuronal cells), testis, prostrate, mammary gland	>Glucose, ≥ galactose, water
	**SGLT2** (*SLC5A2*)	Kidney (cortex/proximal tubules), brain, liver, thyroid, muscle, heart	Glucose, galactose
	**SGLT3** (*SLC5A4/SAAT1*)	(Gluco-sensor) Small intestine, testis, uterus, lung, brain, thyroid, kidney	Glucose, Na^+^ (H^+^)
	**SGLT4** (*SLC5A9*)	Intestine, kidney, liver, brain, lung, trachea, uterus, pancreas	Glucose, mannose, fructose
	**SGLT5** (*SLCA10*)	Kidney (cortex and medulla)	Glucose, galactose
	**SGLT6** (*SLCA11/KST1/SMIT2*)	Brain (neurons), spinal cord, small intestine (ileum and jejunum), Kidney (cortex and medulla), skeletal muscle	Myo-inositol, glucose
	**SMIT** (*SLC5A3*)	Kidney (medulla), choroid plexus blood vessel, thyroid gland, pineal gland, dorsal root ganglion, testes	Myo-inositol, glucose
	**NIS** (*SLC5A5*) (sympoter)	Thyroid, breast, colon, ovary	Iodine (Na^+^/I^−^)
	**SMVT** (*SLC5A6*)	Brain, heart, kidney, lung, placenta	Multivitamins (Biotin, lipoate, pantothenate)
	**CHT** (*SLC5A7/CHT1*)	Spinal cord and medulla (intracellular vesicles)	Choline
